# ED care and Amyloid‐Related Imaging Abnormalities ‐ Impact and Future research

**DOI:** 10.1002/alz.091849

**Published:** 2025-01-09

**Authors:** Neelum T. Aggarwal, Christopher R Carpenter

**Affiliations:** ^1^ Rush Alzheimer’s Disease Center, Chicago, IL USA; ^2^ Rush University Systems for Health, Chicago, IL USA; ^3^ Rush University Medical Center, Chicago, IL USA; ^4^ Mayo Clinic, Rochester, MN USA

## Abstract

With the approval of disease modifying treatments for Alzheimer’s Disease, the practice of dementia care and treatment has changed. Amyloid related imaging changes, are the most common adverse effects of these types of drugs. There is limited guidance across multiple specialties on how to recognize and manage these changes in a dementia patient population. The use of these types of drugs for treatment, impacts multiple stakeholders regarding how they manage and treat dementia patients. ns. Thus the use of these types of drugs, involve multiple stakeholders with different perspectives on how they perceive their role in care and overall treatment plans. In order to optimize collaborative care in the ED setting, recognizing how ARIA may present in a patient who seeks care in the ED, and what types of treatment options are available are needed.

